# Quality of Life in Patients Undergoing Orthognathic Surgery: A Multidimensional Survey

**DOI:** 10.3390/jcm14061923

**Published:** 2025-03-12

**Authors:** Anne-Kathrin Bär, Anna Cäcilia Meier, Olga Konzack, Richard Werkmeister, Nikolaos A. Papadopulos

**Affiliations:** 1 Department of Oral and Maxillofacial Surgery, University Medical Center Mainz, Augustusplatz 2, 55131 Mainz, Germany; 2 Department of Oral and Maxillofacial Surgery, Federal Armed Forces Hospital, Rübenacherstr. 170, 56072 Koblenz, Germany; 3 Private Practice for Oral and Maxillofacial Surgery, Breite Straße 12, 53721 Siegburg, Germany; 4 Department of Plastic Surgery and Hand Surgery, University Hospital Rechts der Isar, Munich Technical University, 81664 Munich, Germany; nikolaos.papadopulos@mri.tum.de; 5 Department of Plastic Surgery and Burns, Alexandroupoli University Hospital, Democritus University of Thrace, 68100 Alexandroupoli, Greece

**Keywords:** orthognathic surgery, quality of life, Rosenberg Self-Esteem Scale, depression, emotionality, patient reported outcomes, survey

## Abstract

**Objectives**: Orthognathic surgery (OGS) is performed to correct dentofacial deformities, improving both function and aesthetics. While prior research suggests positive impacts on quality of life (QoL), self-esteem, and psychosocial well-being, a comprehensive assessment incorporating emotional stability and depressive symptoms remains limited. This study aimed to evaluate the psychological and social effects of OGS, including indication-specific QoL, self-esteem, depression, and emotional stability. **Methods**: A cross-sectional study was conducted using validated questionnaires: the Orthognathic Quality of Life Questionnaire (OQLQ), FACE-Q, Rosenberg Self-Esteem Scale (RSES), Freiburg Personality Inventory (FPI), and Patient Health Questionnaire-9 (PHQ-9). Data were collected from 90 patients who had undergone OGS at a single institution. Results were compared to existing normative data and reference studies on patients before and after OGS and purely aesthetic facial procedures. **Results**: Postoperative patients demonstrated significantly improved QoL across all OQLQ domains. FACE-Q scores indicated high satisfaction with facial appearance and function, aligning with reference studies. The study group exhibited higher self-esteem scores compared to the general population (*p* < 0.001) and showed predominantly balanced emotional stability. However, depressive symptoms were more prevalent in the study group compared to normative data, particularly among male participants (*p* < 0.001). **Conclusions**: The findings suggest that OGS leads to significant improvements in QoL, self-esteem, and emotional stability, reinforcing its positive psychological impact. However, the persistence of depressive symptoms in a subset of patients highlights the need for psychological support during treatment. Given the cross-sectional design, future longitudinal studies are necessary to confirm long-term effects and optimize patient care.

## 1. Introduction

Orthognathic surgery (OGS) corrects skeletal and dental misalignments, enhancing function and appearance. Typically performed on patients with significant dentofacial deformities that cannot be treated with orthodontics alone, OGS has been shown to improve quality of life (QoL) across multiple dimensions [1,2].

The assessment of QoL and patient satisfaction is very complex, as it is a multidimensional concept that encompasses psychological, physical, and social domains subjectively perceived by the individual, and involves the intricate interplay of sensations, expectations, experiences, satisfaction, physical and emotional aspects, and social well-being [3,4,5,6]. Similarly, studies suggest that dental appearance influences perceptions of personality [7], and orthognathic procedures can improve self-confidence, body image and social adjustment [2,8,9,10]. Additionally, studies have underscored differences in the perception of QoL between individuals with and without dentofacial deformities [11,12,13,14].

A comprehensive evaluation must consider aesthetics, function, and psychological and social factors [15]. Validated tools such as the Orthognathic Quality of Life Questionnaire (OQLQ), Oral Health Impact Profile Questionnaire-14 (OHIP-14), and Short Form Health Survey (SF-36) are commonly used [16]. Other methods include interviews and the Rosenberg Self-Esteem Scale [17,18].

Few studies have assessed mental, physical, and social health in OGS patients, including self-esteem, depression, and emotional stability. Patient-Reported Outcome Measures (PROMs) remain underrepresented. Extensive research on QoL in plastic surgery has shown the feasibility of multidimensional assessment tools in different patient populations [19,20,21,22,23,24], which could also be applied to OGS.

This cross-sectional study aimed to evaluate the impact of OGS on QoL, self-esteem, depression, emotional stability, and patient-reported outcomes (PROs) in patients with Class II and III dentofacial deformities. Additionally, the study sought to compare these findings with the existing literature and general population data. The authors hypothesized that OGS positively affects multidimensional quality of life and improves all areas examined, in comparison to the general population and, in particular, in contrast to patients before treatment. A secondary aim was to assess the feasibility and the required effort and burden for patients to plan a future prospective study.

## 2. Materials and Methods

### 2.1. Study Participants

This cross-sectional study included consecutive patients at different postoperative time points who underwent OGS at the Department of Oral and Maxillofacial Surgery, Federal Armed Forces Hospital Koblenz, Germany, between July 2010 and July 2020. Patients were invited to participate voluntarily between May and September 2021. Of the 254 eligible patients, 90 were included. At data collection, all patients had fully recovered and resumed their usual daily, occupational, and social activities.

### 2.2. Inclusion and Exclusion Criteria

Eligible participants had Class II or III skeletal dentofacial deformities. They underwent either single-jaw surgery (bilateral sagittal split osteotomy (BSSO), Le Fort I osteotomy) or bimaxillary surgery (BSSO and Le Fort I). Exclusion criteria included cleft lip and palate, craniofacial syndromes, and facial deformities due to trauma.

### 2.3. Surgical Treatment and Follow-Up

Five experienced surgeons performed the procedures. Referring orthodontists managed pre- and post-operative orthodontic treatment. Titanium miniplate osteosynthesis was used for internal fixation. Routine follow-up concluded with orthodontic appliance removal or 6–9 months post-surgery, typically coinciding with miniplate removal.

### 2.4. Data Collection

Data were collected using six questionnaires: a self-developed, indication-specific questionnaire and five validated tools—OQLQ [25], FACE Q [26,27,28,29], Rosenberg Self-Esteem Scale (RSES) [30], Freiburg Personality Inventory (FPI) [31] and Patient Health Questionnaire-9 (PHQ-9) [32,33]. A similar set of questionnaires has been used in previous studies [20,21,22,23,24].

The assessment time after surgery varied across participants from 6 months to 10 years. This variability was considered in interpreting results, acknowledging that patient-reported outcomes may be influenced by the time elapsed since surgery. After two postal reminders, participants who had not returned their questionnaires were contacted by phone. Of the 154 who initially agreed, 90 returned completed or partially completed questionnaires.

### 2.5. Questionnaires

The self-developed questionnaire assessed demographic and socioeconomic factors, surgical concerns, and treatment satisfaction. The Supplementary Materials provides all questions. In total, the study required participants to answer 91 items, which were derived from both the self-developed and validated questionnaires.

#### 2.5.1. Orthognathic Quality of Life Questionnaire

The OQLQ consists of 22 items across four domains: social aspects, facial aesthetics, oral function, and awareness of dentofacial aesthetics. Each item was rated on a 4-point Likert scale, with higher scores indicating poorer QoL [25]. For the German adaptation, two additional questions were included (OQOL-G) [34].

#### 2.5.2. FACE-Q

The FACE-Q is a comprehensive PROM instrument comprising over 40 independent scales and checklists that evaluate various aspects of facial appearance, quality of life, adverse effects, and patient experience of care [26]. Each scale provides a separate score recorded on a 3- or 4-point Likert scale.

For this study, the following orthognathic-relevant FACE-Q scales were selected: ‘satisfaction with facial appearance overall’ (10 questions), ‘satisfaction with lower face and jawline’ (5 questions) from the ‘satisfaction with appearance’ domain, and ‘social function’ (8 questions) from the ‘quality of life’ domain. The response options for the first two scales range from ‘very dissatisfied’ to ‘very satisfied’ (score ranges: 10–40 and 5–20, respectively). The ‘social function’ scale responses range from ‘definitely disagree’ to ‘definitely agree’ (score range: 8–32). As raw scores are non-linear [35], they were converted to Rasch-transformed scores (0–100), with higher scores indicating greater satisfaction.

To assess changes in patient satisfaction after OGS, results were compared with two reference studies that validated the Chinese and Cantonese FACE-Q versions in Class II and III dentofacial deformity patients pre- and post-OGS [36,37]. Additionally, results were benchmarked against postoperative scores from a large validation study of patients undergoing aesthetic facial procedures [26].

#### 2.5.3. Rosenberg Self-Esteem Scale

The RSES is a 10-item questionnaire measuring self-esteem [30]. Responses are recorded on a 4-point Likert scale from ‘strongly disagree’ to ‘strongly agree’, with half the items positively worded (e.g., life satisfaction) and half negatively worded (e.g., feelings of failure). Higher total scores (0–40) indicate higher self-esteem. The study by Schmitt and Allik provided general population data across 53 nations, with German population norms used as a reference [38].

#### 2.5.4. Freiburg Personality Inventory

The FPI-R(evised) is a psychological assessment tool measuring personality traits across 138 items divided into 12 scales: Life Satisfaction, Social Orientation, Achievement Orientation, Inhibition, Excitability, Aggressiveness, Stress, Physical Complaints, Health Worries, Openness, and additional scales for Extraversion and Emotionality [39]. The Emotionality module, used in this study, evaluates reactions to emotional stimuli and stress. Raw scores were transformed according to sex and age groups to allow normative comparisons [31]. Scores are classified as extremely balanced (1–2), balanced (3–7), or unbalanced/hypersensitive (>7).

#### 2.5.5. Patient Health Questionnaire-9

Depression severity was assessed using the PHQ-9 [32,40]. The nine items align with Diagnostic and Statistical Manual of Mental Disorders (DSM-IV) criteria for major depressive disorder [41], with responses rated from 0 (not at all) to 3 (nearly every day). PHQ-9 scores (0–27) indicate mild (≥5), moderate (≥10), or severe depression (≥15) [32]. Results were compared with normative data from the German population [42].

### 2.6. Statistical Analyses

Data were presented as mean ± standard deviation or frequency (percentage). Analyses were conducted using IBM SPSS Statistics 26.0 (IBM Corp., Armonk, NY, USA). Paired or unpaired *t*-tests were applied for normally distributed data, with statistical significance at *p* < 0.05.

### 2.7. Ethical Considerations

Written informed consent was obtained from all participants in accordance with the Declaration of Helsinki. The study was approved by the local ethics committee (No. 2021-15595_1).

## 3. Results

### 3.1. Self-Developed, Indication-Specific Questionnaire

A total of 90 subjects (44 female, 42 male, and 4 non-binary) participated in the study (dropout rate 64.6%). The mean age was 33.3 ± 10 years (range: 20–62 years). Dentofacial deformities were corrected by bimaxillary jaw surgery (*n* = 18, 20%), Le Fort I (*n* = 6, 7%), or BSSO (*n* = 66, 73%), with the majority involving mandibular setback (*n* = 38, 42%).

For most patients, treatment-related stress met expectations (*n* = 55, 61.1%). It was lower than expected at 22.2% (*n* = 20), while 16.7% (*n* = 15) experienced a higher-than-expected burden. The most commonly reported difficulties were intermaxillary fixation (*n* = 44, 48.9%), nasogastric tube irritation (*n* = 43, 47.8%), and swelling (*n* = 40, 44.4%). Long-term complications included reduced jaw opening (*n* = 13, 14.4%).

Postoperatively, patients reported the most significant improvements in biting and chewing (*n* = 75, 83.3%), facial profile (*n* = 57, 63.3%), reduced temporomandibular joint pain (*n* = 24, 26.7%), and improved pronunciation (*n* = 12, 13.3%). Overall, 74 patients (84.1%) were very or somewhat satisfied with the surgical outcome, and 63 patients (71.5%) indicated they would “definitely” or “probably” undergo surgery again. The full results are provided in the Supplementary Materials.

### 3.2. Orthognathic Quality of Life Questionnaire

Table 1 presents the mean OQLQ scores for the study group compared to pre-treatment patients with dentofacial deformities from Bock et al.’s reference study. The study group’s scores fell within the median range, suggesting moderate impairment in several QoL domains, including oral function, social aspects, awareness of dentofacial aesthetics, and facial aesthetics.

Significant differences were observed between the groups in all four domains. Following OGS, patients showed improved oral function (MD 4.3), social aspects of dentofacial deformity and facial aesthetics (MD 3.0 for each), and awareness of dentofacial aesthetics (MD 1.9) (all *p* < 0.05).

### 3.3. FACE-Q

Table 2 presents the mean scores for selected FACE-Q scales in the study group, comparing them to pre- and post-surgical values from reference studies. The study group consistently exhibited higher scores post-surgically compared to pre-surgical reference populations across all measured domains.

For Facial Appearance Overall, the study group achieved a mean post-surgical score of 67.4 ± 19.0, which was similar to the post-surgical values reported in the reference studies (Su et al.: 68.1 ± 20.4, Tan et al. Class II: 67.7 ± 20.1, Class III: 66.9 ± 18.0).

In the Social Self-Confidence domain, the study group reported a mean score of 62.7 ± 20.6, which was higher than the post-surgical score from Su et al. (56.7 ± 23.6) but lower than the post-surgical values reported by Tan et al.’s Class II (68.8 ± 18.8) and Class III (71.6 ± 19.6) groups. The Klassen et al. (2015) validation study, which focused on patients undergoing purely aesthetic facial procedures, reported a post-surgical mean of 68.1 ± 19.2 [26].

Regarding Lower Face and Jawline, the study group reported a mean score of 71.5 ± 22.6, aligning with Su et al. (72.3 ± 21.0) and Tan et al. (Class II: 69.7 ± 20.9, Class III: 70.3 ± 20.4). Klassen et al. (2014) found a lower mean of 60.0 ± 26.0 in patients undergoing purely aesthetic procedures, indicating that orthognathic surgery patients generally report higher satisfaction with the lower face and jawline compared to aesthetic surgery patients [28].

### 3.4. Rosenberg Self-Esteem Scale

The study group reported significantly higher self-esteem scores compared to the normative population (34.8 ± 5.5 vs. 31.7 ± 4.7, *p* < 0.001) [38]. This result indicates a notably higher self-confidence among study participants (Table 3).

### 3.5. Freiburg Personality Inventory

The study group had a mean score of 3.6 ± 3.4 on the Emotionality scale of the FPI, which was significantly lower than the normative data from the general German population (5.4 ± 3.7, *p* < 0.001) [31] (Table 3).

**Figure 1:**
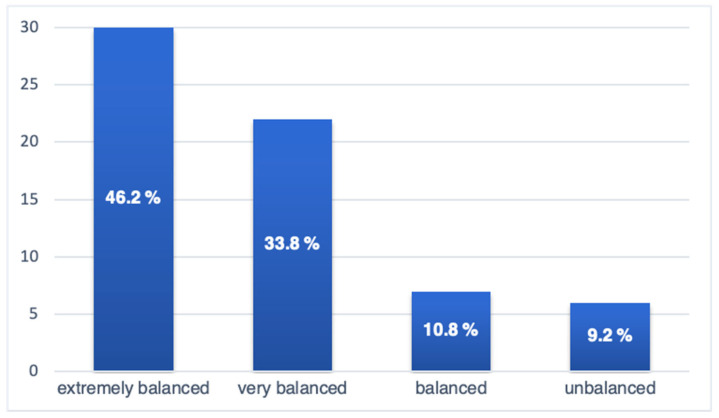
Emotionality scales of the Freiburg Personality Inventory. Percentage and absolute distribution of participants within each emotional stability category in the study group.

Based on categorized emotional stability levels, 46.2% (*n* = 30) of the participants were classified as extremely balanced, 33.8% (*n* = 22) as very balanced, 10.8% (*n* = 7) as balanced, and 9.2% (*n* = 6) as unbalanced (Figure 1).

These findings indicate that the majority of the study group demonstrated a high degree of emotional stability, with a smaller proportion exhibiting mild to moderate emotional instability.

### 3.6. Patient Health Questionnaire-9

The study group had a mean PHQ-9 score of 4.2 ± 4.7, which was significantly higher than the normative data from the German population (2.9 ± 3.5, *p* < 0.001) [42] (Table 3).

When stratified by gender, male participants in the study group had a mean PHQ-9 score of 4.6 ± 5.1, which was significantly higher than the normative male population (2.7 ± 3.5, *p* < 0.001). In contrast, female participants had a mean score of 4.0 ± 4.4, which was higher than the normative female population (3.1 ± 3.5) but did not reach statistical significance (*p* = 0.111).

Further classification of depression severity within the study group showed that 37 participants had minimal symptoms, 17 had mild symptoms, and 1 participant had moderate symptoms. Additionally, six individuals met the criteria for major depressive disorder, with a predominance among male participants.

## 4. Discussion

This study aimed to evaluate the effects of OGS on patients with dentofacial deformities, focusing on QoL, self-esteem, depression, emotional stability, and PROs related to external appearance. The results indicate improvements across these domains, consistent with prior research [8,9]. However, due to the inherent limitations of cross-sectional design, these findings should be interpreted with caution.

A multidimensional approach was employed, utilizing validated questionnaires (OQLQ, FACE-Q, RSES, FPI, and PHQ-9) to assess various psychosocial factors, including PROs through the FACE-Q, which provided valuable insights into subjective perceptions of surgical outcomes. However, the timing of data collection varied significantly among participants, ranging from 6 months to 10 years postoperatively. This variability may have influenced patient-reported satisfaction and recall accuracy, particularly in those who had undergone surgery several years prior. Future studies should consider stratifying results by defining postoperative time points to better understand how psychological adaptation and satisfaction evolve over time.

Preoperatively, patients exhibited moderate impairments in several QoL aspects, as reflected in OQLQ scores [2,34]. Following surgery, significant improvements were observed in all OQLQ domains, aligning with findings from systematic reviews [2,16]. The FACE-Q results confirmed these trends, showing increased satisfaction with facial appearance, lower face and jawline, and social function post-OGS [36,37]. Reference studies indicate that patients with lower preoperative satisfaction tend to experience greater postoperative improvements in facial aesthetics and function [26,28]. The postoperative results of the study group align with these findings.

The study group exhibited significantly higher self-esteem scores than the general population [38]. This finding supports previous research showing that OGS enhances self-confidence, particularly in patients with Class III malocclusions [43,44]. Emotional stability, assessed via the FPI, indicated a predominantly balanced disposition among participants, with a significant proportion classified as emotionally stable. This suggests a positive psychological impact of OGS beyond physical correction.

Despite improvements in self-esteem and emotional stability, depressive symptoms were more prevalent in the study group compared to the normative population [42]. These findings align with studies reporting that depressive symptoms persist post-OGS [45,46,47], suggesting that underlying psychological factors may influence mental health outcomes beyond surgical intervention. Although the mean depression score was within the minimal range, male participants had significantly higher PHQ-9 scores than their normative counterparts (*p* < 0.001), whereas female participants did not show a significant difference. This gender difference warrants further exploration, as societal expectations, coping mechanisms, and preoperative psychological conditions may contribute to these findings. Future studies should aim to clarify the underlying causes of these disparities and assess whether targeted preoperative psychological support could mitigate depressive tendencies in male patients.

This study has several limitations, including its cross-sectional design, which precludes causality assessment. The involvement of five experienced surgeons ensured a standardized surgical approach, while the variability among referring orthodontists managing pre- and post-operative orthodontic treatment could have influenced treatment consistency. The heterogeneous nature of the sample must be considered when interpreting the results. Patients differed in their social, emotional, and psychological backgrounds, and their postoperative recovery times varied significantly. These factors add to the study’s complexity but also introduce potential confounders. Future studies should aim for more stratified analyses or control for these variables to improve the generalizability of findings. Another major limitation was the high dropout rate (64.6%), likely due to the questionnaire burden (91 items). The time required to complete the surveys (45–60 min) may have contributed to nonresponse bias. Future research should explore alternative data collection strategies, such as shorter, adaptive electronic surveys, to improve participation rates and data completeness while maintaining measurement accuracy. Additionally, prospective longitudinal studies are needed to assess long-term psychosocial outcomes and provide deeper insights into the sustained impact of surgical interventions over time.

Overall, this study highlights the multifaceted impact of OGS on patients’ psychological and social well-being. The findings support the hypothesis that OGS leads to significant improvements in QoL, self-esteem, and emotional stability, except for depressive tendencies. Incorporating PROMs adds to the study’s clinical relevance, helping surgeons and psychologists optimize patient care. Future research should focus on identifying factors influencing persistent depressive symptoms and optimizing support strategies for patients undergoing OGS.

## 5. Conclusions

This study provides a comprehensive assessment of the psychological and social effects of OGS on patients with dentofacial deformities. The findings demonstrate significant improvements in QoL, self-esteem, and emotional stability following surgery. Additionally, reference studies indicate that patients with lower preoperative satisfaction tend to experience greater postoperative improvements in facial aesthetics and function, aligning with the postoperative results observed in the study group. However, the persistence of depressive symptoms in a subset of patients suggests the need for further psychological support during the treatment process.

While the study reinforces the positive impact of OGS, its cross-sectional nature and sample limitations highlight the necessity of future longitudinal studies to better understand long-term outcomes. Reducing patient burden in questionnaire-based studies and improving sample representativeness should be key priorities in future research. These insights are essential for optimizing patient care, refining treatment protocols, and enhancing the overall well-being of individuals undergoing OGS.

## Figures and Tables

**Table 1:** Comparison of Orthognathic Quality of Life Questionnaire (OQLQ) scores to those of patients with dentofacial deformities before orthodontic and surgical treatment.

	Bock et al. [34] Pre-Treatment *n* = 50		Study Group Post-Treatment *n* = 90			
	Mean	(SD)	Mean	(SD)	MD	*p*-Value
Oral function (0–20)	12.1	(5.6)	7.8	(3.2)	4.3	<0.001
Social (0–32)	14.7	(8.9)	11.7	(5.1)	3.0	0.011
Awareness (0–16)	9.9	(3.8)	8.0	(3.1)	1.9	0.003
Facial esthetics (0–20)	11.5	(5.5)	8.5	(3.1)	3.0	<0.001

OQLQ, Orthognathic Quality of Life Questionnaire; SD, standard deviation; MD, mean difference; Statistically significant *p*-value < 0.05, independent-samples *t*-test.

**Table 2:** FACE-Q Scores: Study Data Compared to Pre- and Postsurgical Data from Reference Studies (Rasch Model).

Facial Appearance Overall	Sample Size (*n*)	Pre-Surgical Mean ± SD	Post-Surgical Mean ± SD
Study Group	89	ND	67.4 ± 19.0
Su et al. [36]	44	44.5 ± 19.4	68.1 ± 20.4
Tan et al. [37]			
Class II	23	41.1 ± 8.6	67.7 ± 20.1
Class III	111	43.6 ± 11.8	66.9 ± 18.0
**Social Self-Confidence**	**Sample Size (*n*)**	**Pre-Surgical** **Mean ± SD**	**Post-Surgical** **Mean ± SD**
Study Group	89	ND	62.7 ± 20.6
Su et al. [36]	44	42.9 ± 21.7	56.7 ± 23.6
Tan et al. [37]			
Class II	23	47.9 ± 12.2	68.8 ± 18.8
Class III	111	51.3 ± 15.6	71.6 ± 19.60
Klassen et al. [26] Validation study	264	58.8 ± 18.7	68.1 ± 19.2
**Lower Face and Jawline**	**Sample Size (*n*)**	**Pre-Surgical** **Mean ± SD**	**Post-Surgical** **Mean ± SD**
Study Group	89	ND	71.5 ± 22.6
Su et al. [36]	44	35.1 ± 26.7	72.3 ± 21.0
Tan et al. [37]			
Class II	23	31.2 ± 15.4	69.7 ± 20.9
Class III	111	37.6 ± 15.7	70.3 ± 20.4
Klassen et al. [28] Validation study	97	44.0 ± 24.0	60.0 ± 26.0

Values of 30 correspond to “very dissatisfied”; values of 100 correspond to “very satisfied.” ND, no data available or data cannot be separated.

**Table 3:** Self-Esteem, Emotional Stability, and Depression: Study Data compared to normative data from the German population.

Self-Esteem (Rosenberg Self-Esteem Scale, RSES)	Sample Size (*n*)	Mean ± SD (Range)
Study Group	88	34.8 ± 5.5 (11–40)
Normative Data [38]	782	31.7 ± 4.7 (10–40)
*p*-value		<0.001
**Emotional Stability (Freiburg Personality Inventory, FPI)**	**Sample Size (*n*)**	**Mean ± SD**
Study Group	89	3.6 ± 3.4
Normative Data [31]	2579	5.4 ± 3.7
*p*-value		<0.001
**Depression (Patient Health Questionnaire-9, PHQ-9)**	**Sample Size (*n*)**	**Mean ± SD (Range)**
Study Group (Total)	80	4.2 ± 4.7 (0–22)
Normative Data [42] (Total)	5018	2.9 ± 3.5
*p*-value		<0.001
Study Group (Female)	40	4.0 ± 4.4 (0–22)
Normative Data [42] (Female)	2692	3.1 ± 3.5
*p*-value		0.111
Study Group (Male)	40	4.6 ± 5.1 (0–17)
Normative Data [42] (Male)	2326	2.7 ± 3.5
*p*-value		<0.001

Statistically significant *p*-value < 0.05, unpaired sample *t*-test.

## Data Availability

The raw data supporting the conclusions of this article will be made available by the authors on request.

## Supplementary Materials

The following supporting information can be downloaded at: https://www.mdpi.com/article/10.3390/jcm14061923/s1, Table S1: Gender Distribution. Table S2. Age Distribution. Table S3. Results of the Self-Developed, Indication-Specific Questionnaire.

## Abbreviations

The following abbreviations are used in this manuscript:

QoL	Quality of Life
OQLQ	Orthognathic Quality of Life Questionnaire
OHIP-14	Oral Health Impact Profile–14
SF-36	Short Form Health Survey
PROM(s)	Patient-Reported Outcome Measure(s)
PRO(s)	Patient-Reported Outcome(s)
BSSO	bilateral sagittal split osteotomy
RSES	Rosenberg Self-Esteem Scale
FPI	Freiburg Personality Inventory
PHQ-9	Patient Health Questionnaire-9
